# Clinical implications of carcinoembryonic antigen distribution in serum exosomal fraction—Measurement by ELISA

**DOI:** 10.1371/journal.pone.0183337

**Published:** 2017-08-17

**Authors:** Shozo Yokoyama, Akihiro Takeuchi, Shunsuke Yamaguchi, Yasuyuki Mitani, Takashi Watanabe, Kenji Matsuda, Tsukasa Hotta, John E. Shively, Hiroki Yamaue

**Affiliations:** 1 Second Department of Surgery, Wakayama Medical University, School of Medicine, Wakayama, Japan; 2 Department of Molecular Immunology, Beckman Research Institute of the City of Hope, Duarte, California, United States of America; Gustave Roussy, FRANCE

## Abstract

**Background:**

Serum exosomal proteins have great potential as indicators of disease status in cancer, inflammatory or metabolic diseases. The association of a fraction of various serum proteins such as carcinoembryonic antigen (CEA) with circulating exosomes has been debated. The establishment of a method to measure the exosomal fraction of such proteins might help resolve this controversy. The use of enzyme-linked immunosorbent assays (ELISAs) to measure serum exosomal molecules, for example CEA, is rare in research laboratories and totally absent in clinical biology. In this study, we optimized a method for assessment of serum exosomal molecules combining a treatment by volume-excluding polymers to isolate the exosomes, their subsequent solubilization in an assay buffer and ELISA.

**Methods:**

One hundred sixteen consecutive patients with colorectal cancer were enrolled for this study between June 2015 and June 2016 at Wakayama Medical University Hospital (WMUH). Whole blood samples were collected from patients during surgery. Exosomes were isolated using the ExoQuick reagent, solubilized in an assay buffer and subjected to CEA detection by ELISA. The procedure of serum exosome isolation and the formulation of the assay buffer used for the ELISA were optimized in order to improve the sensitivity and specificity of the assay.

**Results:**

A five-fold increase in the concentration of the exosomes in the assay buffer (using initial serum volume as a reference) and the addition of bovine serum albumin (BSA) resulted in more accurate measurements of the serum exosomal CEA. The thawing temperature of frozen serum samples before exosome extraction was also optimized. A validation study that included one hundred sixteen patients with colorectal cancer demonstrated that serum exosomal CEA from samples thawed at 25°C exhibited a better AUC value, sensitivity, and specificity as well as a more correct classification than serum CEA.

**Conclusions:**

We optimized an easy and rapid detection method for assessment of serum exosomal CEA. The thawing temperature of frozen serum prior to exosome extraction, the formulation of the assay buffer used for exosome solubilization and the concentration of the exosomes in this buffer were fine-tuned to enable the appropriate and accurate measurement of serum exosomal CEA.

## Introduction

An exosome is a small vesicle, measuring 40–150 nm in size [1], and is characterized based on its origin [2]. Various cell types are able to generate and release exosomes [3–5], and exosomes reflect the characteristics of their parent cells [6–8]. Furthermore, an exosome can transfer various signals via proteins, lipids, DNA, RNA, and microRNA [9–11]. Therefore, it has been speculated that the accurate measurement of serum exosomal markers may provide a great deal of information regarding diseases such as cancer [6, 12, 13] and inflammation [14] and/or metabolic and cardiovascular diseases [15]. The use of enzyme-linked immunosorbent assays (ELISAs) to measure serum exosomal molecules is neither common nor clinically available. To address these issues, we investigated the feasibility of measuring serum molecules by ELISA and volume-excluding polymers for exosome isolation. The goal is to provide a simple and rapid method for performing such measurements for both clinical and research applications.

Exosomal miR-19a [16] and exosomal CD9 and CD147 [17] are diagnostic and predictive markers for colorectal cancer, whereas exosomal carcinoembryonic antigen (CEA) has not yet been investigated in this context. Because serum CEA is used clinically as a marker worldwide, we selected CEA as a target molecule in this study, although it has certain limitations. CEA is used to monitor the recurrence of colorectal cancer but not for early detection [18]. Serum CEA is also associated with high false-positive rates [8], leading to unnecessary examinations following curative surgical resection of colorectal cancer. Serum CEA includes secreted and cleaved CEA as well as exosomal CEA. Normal colonic mucosa secretes CEA [19]. If cancer cells release more exosomes [17] than normal cells, the measurement of serum exosomal CEA may enable the discrimination between normal and morbid conditions. Moreover, clinicians and surgeons need a tumor marker that can be used to discriminate between the presence and absence of distant metastasis before and after curative resection.

In the present study, we optimized a method to measure serum exosomal CEA by utilizing volume-excluding polymers and ELISA, and we examined whether serum exosomal CEA can better predict the existence of distant metastasis than serum CEA.

## Materials and methods

### Participants

We enrolled 116 consecutive patients treated for colorectal cancer between June 2015 and June 2016 at Wakayama Medical University Hospital and 8 normal healthy subjects. Written informed consent was obtained from all subjects. The mean age of the patients was 68.9 years (range 42–92), and there were 66 males and 50 females. Based on the TNM classification system, 39, 22, 36, and 19 patients had colorectal carcinoma of stages I, II, III, and IV, respectively. All patients underwent surgery, during which whole blood samples were collected. The current study was approved by the Human Ethics Review Committee of Wakayama Medical University Hospital. All experiments were performed in accordance with relevant guidelines and regulations.

### Sample preparation and ELISA

Whole blood was collected into serum-separating tubes and then centrifuged at 1000 ×*g* for 10 min at 4°C. Serum was distributed in 250-μl aliquots into Eppendorf tubes and frozen at –20°C until analysis. Frozen serum samples were thawed at 4, 25, or 37°C for 30 min. A total of 63 μl of ExoQuick Exosome Precipitation Solution (System Biosciences, Palo Alto, CA, USA) was added to each thawed 250-μl serum sample, which was then incubated at room temperature for 30 min. The serum/ExoQuick mixture was centrifuged at 1500 ×*g* for 30 min at 4°C. The supernatant was aspirated, and the exosome pellet was resuspended using water or 1% bovine serum albumin (BSA). A CEA (human) ELISA kit (Abnova, Taipei City, Taiwan) was used in our assay.

### Western blot analysis for CD63

To evaluate whether thawing temperature at 4, 25, or 37°C modulates the yield and purity of exosome, western blotting for CD63 as exosomal marker was performed. Frozen serum samples from normal healthy subject were thawed at 4, 25, or 37°C for 30 minutes. After exosome isolation by using ExoQuick Exosome Precipitation Solution, protein extraction was performed with RIPA lysis buffer (Santa Cruz Biotechnology, Paso Robles, CA, USA). The samples were subjected to sodium dodecyl sulphate-polyacrylamide gel electrophoresis. In each lane, 10 μl of each sample was loaded and separated in a precast polyacrylamide gel (Thermo Fisher Scientific, Waltham, MA, USA). Proteins were then electrotransferred onto PVDF membrane (Bio-Rad, Hercules, CA, USA). After blocking the membrane with 5% electrophoresis-grade nonfat milk, primary and secondary antibodies were incubated for 60 min each in a 5% milk solution. Immune complexes were visualized by incubating the membranes with an HRP-conjugated anti-mouse antibody using the ECL detection reagent (GE Healthcare, Little Chalfont, Buckinghamshire, UK). The primary antibody was mouse monoclonal CD63 antibody (Ts63, diluted at 1:250, Thermo Fisher Scientific). The secondary antibody was sheep anti-mouse IgG conjugated to HRP (diluted 1:1000, GE Healthcare).

### Optimization of pre-analytical conditions

To optimize measurement of serum exosomal CEA, the effects of concentration of the samples, formulation of the solvent, and thawing temperatures for frozen samples were evaluated. Samples from eight patients and eight normal healthy subjects were used. Each patient’s exosome pellet was resuspended using 250 μl of Milli-Q water (1×), 50 μl of Milli-Q water (5×), 250 μl of 1% BSA (1×) or 50 μl of 1% BSA (5×). Frozen serum samples from the same participant were thawed at 4, 25, and 37°C. An EXOCET Exosome Quantitation Kit(System Biosciences) and a CEA (human) ELISA kit (Abnova) were used for the quantitation of each exosome sample and the measurement of exosomal CEA concentrations, respectively.

### Comparison of serum exosomal CEA at various thawing temperatures for diagnosing presence of distant metastasis

Because serum CEA values are used to diagnose suspected colorectal cancer recurrence, serum exosomal CEA concentrations at each temperature were evaluated as a potential diagnostic marker indicating the existence of distant metastasis. Forty-eight patients were enrolled in this part of the study. Frozen serum samples from the same patient were thawed at 4, 25, and 37°C. Accuracy, sensitivity, specificity, and correct classification were evaluated.

### Validation study comparing serum exosomal CEA and serum CEA values for diagnosing presence of distant metastasis

A validation study was conducted using samples from all 116 colorectal cancer patients. Frozen serum samples were thawed at an appropriate temperature obtained from the results based on the comparison described in the previous section. Accuracy, sensitivity, specificity, and correct classification were compared for serum exosomal CEA and serum CEA.

### Statistical analysis

The accuracy of serum exosomal CEA was measured using the area under the ROC curve. Sensitivity, specificity, and correct classification were also calculated from the curve. Statistical calculations were performed using the STATA software program, version 13 (StataCorp, College Station, TX, USA). *P*-value < 0.05 was considered to be statistically significant.

## Results

### Solvent and concentration adjustments permit the measurement of serum exosomal CEA

**Fig 1:**
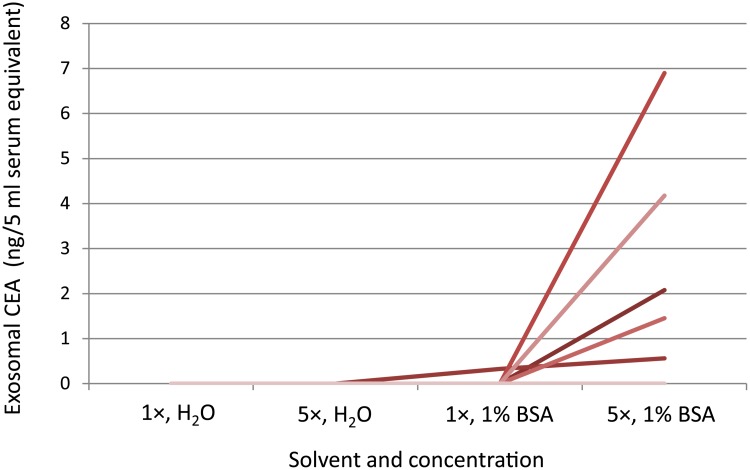
Relationships between exosomal CEA concentration and two solvents at different concentrations. The results obtained for samples dissolved in 250 μl of Milli-Q water (H_2_O) (1×), 50 μl of Milli-Q water (5×), 250 μl of 1% BSA (1×), or 50 μl of 1% BSA (5×) for the same patient.

Exosomal proteins such as CEA are present in small amounts, so high protein concentrations may be needed for their detection using ELISA. The present study demonstrated that ELISA could not detect a 1× CEA sample dissolved in Milli-Q water. Even when the exosome concentration was increased by a factor of five, serum exosomal CEA remained undetectable (Fig 1). This inability to detect serum exosomal CEA may be attributable to not only a low CEA concentration but also the use of water as a solvent. When 1% BSA was used as a solvent, serum exosomal CEA concentrations could be measured (Fig 1). A five-fold increase in the concentration and the use of 1% BSA as a solvent are therefore required for the detection of serum exosomal CEA.

### Thawing temperature of frozen samples alters serum exosomal CEA values without changing exosome quantity

**Fig 2:**
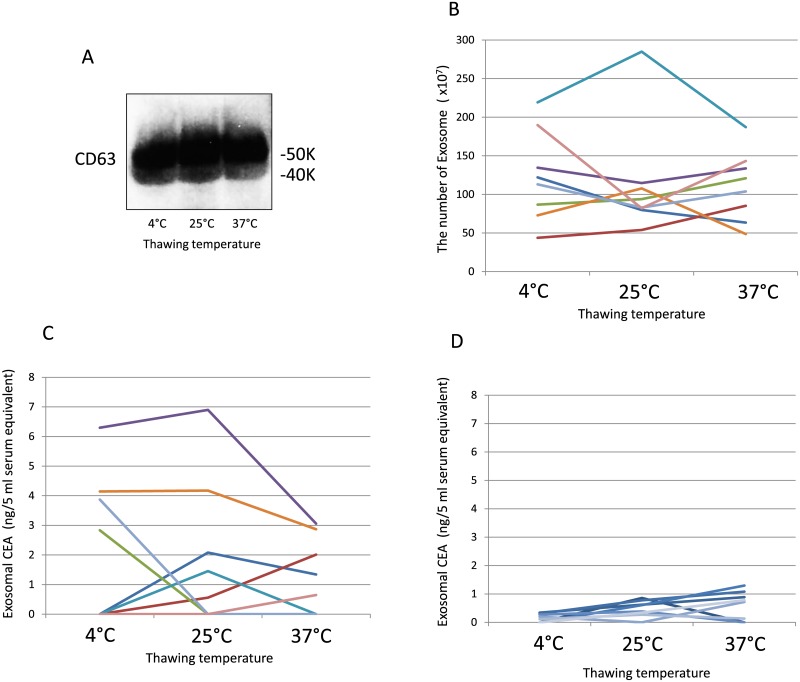
Effects of thawing temperature on serum exosomal CEA values and quantity. A: Western blotting for CD63 of exosome from frozen serum samples from normal healthy subject that were thawed at 4, 25, and 37°C. B: Each color indicates the results obtained from exosome quantitation for frozen serum samples from the same patient that were thawed at 4, 25, and 37°C. C: Each color indicates exosomal CEA values measured using ELISA for frozen serum samples from the same patient that were thawed at 4, 25, and 37°C. The same colors in B and C correspond to the same patients. D: Exosomal CEA values for frozen serum samples from eight normal healthy subjects that were thawed at 4, 25, and 37°C.

The efficacy of volume-excluding polymers depends on the hydrophilicity or hydrophobicity of the substrate. We hypothesized that the thawing temperature of frozen serum alters the hydrophilicity or hydrophobicity of exosomes and CEA molecules. Based on this hypothesis, frozen serum was thawed before exosome isolation with ExoQuick reagent. The yield in western blots and quantity of exosomes did not differ among the various thawing temperatures (4, 25, and 37°C; Fig 2A and 2B). However, exosomal CEA values measured using ELISA differed among temperatures (Fig 2C), indicating that the thawing temperature may alter the antigenicity of the CEA molecule. To exclude the possibility of false-positive results, samples from eight normal healthy subjects were examined. All exosomal CEA values measured at different thawing temperature using ELISA were low, and not differed among the temperatures (Fig 2D).

### Thawing temperature of frozen samples alters the accuracy of serum exosomal CEA values for diagnosis of distant metastasis in colorectal cancer patients

**Fig 3:**
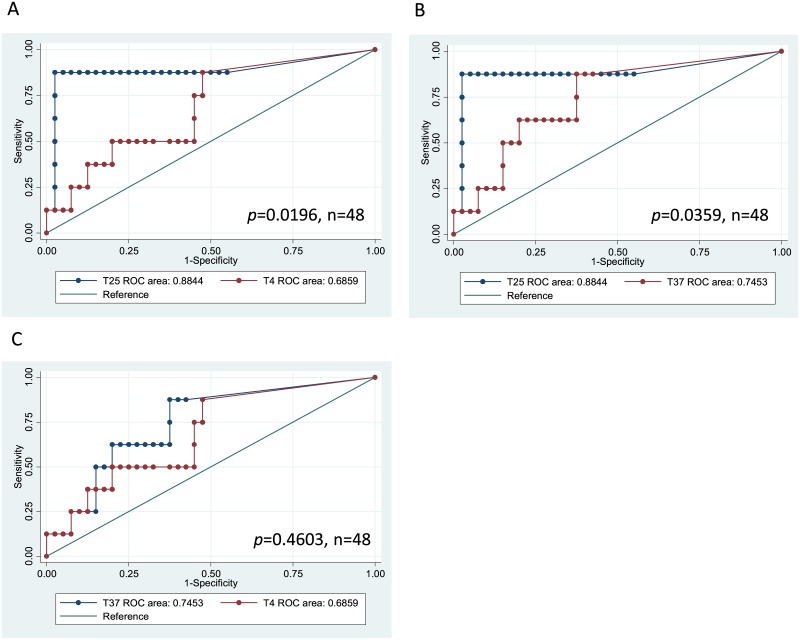
ROC curves for the presence of distant metastasis of colorectal cancer for serum exosomal CEA samples thawed at 4, 25, or 37°C. Data represent samples from 48 patients. A: Serum exosomal CEA samples thawed at 25 (T25) or 4°C (T4). B: Serum exosomal CEA samples thawed at 25 or 37°C. C: Serum exosomal CEA samples thawed at 4 or 37°C.

**Fig 4:**
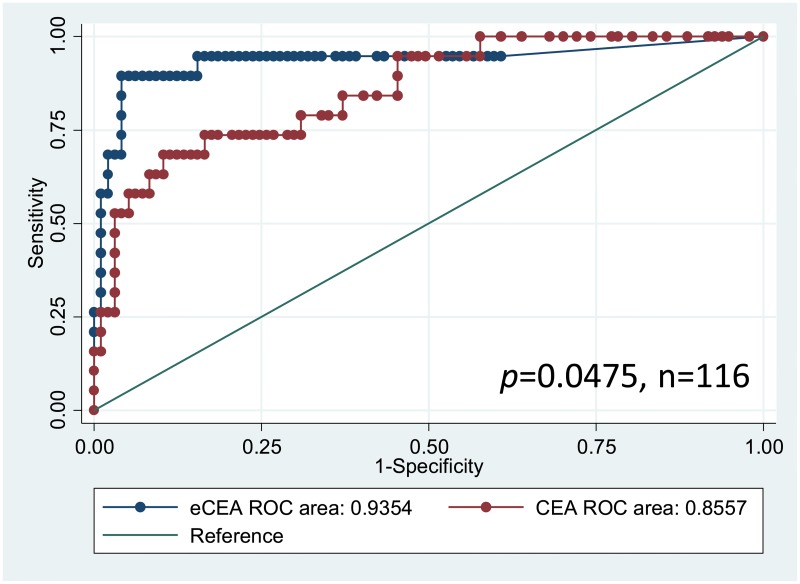
Serum exosomal CEA and serum CEA for diagnosis of distant metastasis in colorectal cancer patients. ROC curves for the presence of distant metastasis in colorectal cancer patients based on serum exosomal CEA samples thawed at 25°C and serum CEA from 116 patients with colorectal cancer; eCEA: serum exosomal CEA.

Thawing temperature affected the measured values of serum exosomal CEA. Samples thawed at 25°C exhibited a significantly better AUC value for diagnosis of distant metastasis than samples thawed at 4 or 37°C (Fig 3A and 3B). AUC values were similar between samples thawed at 4 and 37°C (Fig 3C). The use of appropriate cut-off values with 25°C thawing temperature produced better sensitivity and specificity and more accurate classification (Table 1). A validation study that included all 116 colorectal cancer patients verified that samples thawed at 25°C exhibited a better AUC value, sensitivity, and specificity as well as more accurate classification than serum CEA (Fig 4, Table 2).

**Table 1:** Sensitivity, specificity, and correct classification for the existence of distant metastasis for serum exosomal CEA samples thawed at 4, 25, or 37°C and serum CEA samples from 48 patients.

Thawing temp.	Cut-off point	Sensitivity	Specificity	Correctly classified
4°C	≥2.634	50.00%	80.00%	75.00%
25°C	≥2.29	87.50%	97.50%	95.83%
37°C	≥1.973	62.50%	80.00%	77.08%

**Table 2:** Sensitivity, specificity, and correct classification of the presence of distant metastasis for serum exosomal CEA samples thawed at 25°C and serum CEA samples from 116 patients with colorectal cancer.

	Cut-off point	Sensitivity	Specificity	Correctly classified
Exosomal CEA (25°C)	≥2.29	89.47%	95.88%	94.83%
Serum CEA	≥5	78.95%	69.07%	70.69%

## Discussion

In the present study, we showed that measurement of serum exosomal CEA required a high concentration and the appropriate formulation of the solvent. When Milli-Q water was used as a solvent for the exosome sample serum exosomal CEA could not be detected by ELISA, whereas when 1% BSA was used it was able to be measured. The use of 1% BSA as a solvent is therefore required for the detection of serum exosomal CEA. Notably, 1% BSA, which is typically used as a blocking buffer for ELISA, may alter the antigen—antibody reaction or interactions among exosomes. Further investigations are required to address how 1% BSA contributes to the detection of CEA.

CEA is a glycoprotein and is generally hydrated. The thawing temperature for frozen samples may change the hydration of CEA, which affects antibody responses against the antigen. Therefore, different thawing temperatures were examined. Exosome quantities did not differ among thawing temperatures, but exosomal CEA values measured using ELISA differed among thawing temperatures. Therefore, thawing temperature may affect the antigenicity of CEA by affecting the dehydration status. Additional molecular research is required to address this variation.

It is important for clinicians and surgeons to discriminate between local and systemic disease status of patients with colorectal cancer, because each calls for different therapies, such as surgery, chemotherapy, and radiotherapy. The accurate diagnosis of distant metastasis in colorectal cancer can let clinicians and surgeons devise a proper treatment plan. Moreover, the accurate diagnosis of distant metastasis also benefits patients without recurrence of colorectal cancer, because a false-positive test result leads to improper examinations. The present study demonstrated that measurements of serum exosomal CEA could increase the rate of correct diagnosis and could allow discrimination between the presence and absence of distant metastasis. Serum exosomal CEA is a promising tumor marker for diagnosis of metastatic colorectal cancer.

This method may be applicable for other proteins and glycoproteins. However, this study had certain limitations. The selected concentration, solvent, and temperature are not common across all exosomal proteins and glycoproteins and must be adjusted to the selected exosomal glycoprotein. Moreover, because our data are derived with respect to a specific exosome isolation reagent and ELISA kit, each parameter should be adjusted for the selected reagent and kit. We used a ready-made ExoQuick reagent and a CEA (human) ELISA kit. Neither the reagent nor the antibodies were prepared for the detection of exosomal markers. Exosome isolation reagents should be developed for specific exosomal markers. The measurement range for the CEA (human) ELISA kit used in this study was 0 to 120 ng/ml, which is unsuitable for detecting exosomal CEA, because the cut-off value for exosomal CEA was approximately 2 ng/ml in the current study. Appropriate exosome isolation reagents and ELISA kits specific for exosomal markers need to be improved and established for appropriate applications involving exosomal proteins and glycoproteins.

In conclusion, we optimized a simple and rapid detection method for exosomal glycoproteins such as CEA. The concentration of the exosome sample, the formulation of the solvent for the exosomes, and the thawing temperature of frozen serum before extraction were fine-tuned to enable the appropriate and accurate measurement of exosomal CEA.
